# GeneSrF and varSelRF: a web-based tool and R package for gene selection and classification using random forest

**DOI:** 10.1186/1471-2105-8-328

**Published:** 2007-09-03

**Authors:** Ramón Diaz-Uriarte

**Affiliations:** 1 Statistical Computing Team, Structural Biology and Biocomputing Programme, Spanish National Cancer Center (CNIO), Melchor Fernández Almagro 3, Madrid, 28029, Spain

## Abstract

**Background:**

Microarray data are often used for patient classification and gene selection. An appropriate tool for end users and biomedical researchers should combine user friendliness with statistical rigor, including carefully avoiding selection biases and allowing analysis of multiple solutions, together with access to additional functional information of selected genes. Methodologically, such a tool would be of greater use if it incorporates state-of-the-art computational approaches and makes source code available.

**Results:**

We have developed GeneSrF, a web-based tool, and varSelRF, an R package, that implement, in the context of patient classification, a validated method for selecting very small sets of genes while preserving classification accuracy. Computation is parallelized, allowing to take advantage of multicore CPUs and clusters of workstations. Output includes bootstrapped estimates of prediction error rate, and assessments of the stability of the solutions. Clickable tables link to additional information for each gene (GO terms, PubMed citations, KEGG pathways), and output can be sent to PaLS for examination of PubMed references, GO terms, KEGG and and Reactome pathways characteristic of sets of genes selected for class prediction. The full source code is available, allowing to extend the software. The web-based application is available from . All source code is available from Bioinformatics.org or The Launchpad. The R package is also available from CRAN.

**Conclusion:**

varSelRF and GeneSrF implement a validated method for gene selection including bootstrap estimates of classification error rate. They are valuable tools for applied biomedical researchers, specially for exploratory work with microarray data. Because of the underlying technology used (combination of parallelization with web-based application) they are also of methodological interest to bioinformaticians and biostatisticians.

## Background

Patient classification and gene selection related to classification are common uses of microarray data (e.g., review and references in [1]), but statistically rigorous and user-friendly tools for gene selection in the context of class prediction are rare. Such a tool should address two major issues. First, it should provide unbiased estimates of the prediction error rate of the procedure. Most users are by now aware of "selection bias", as originally reported in [2,3], but bias caused by trying different methods and/or sets of genes, and choosing the one with the smallest cross-validated error rate [4] is still not widely recognized. In this later case we need a nested [4] or double or full cross-validation [5] to estimate the error rate of the rule or procedure. Second, we need to assess the so called multiplicity (or lack of uniqueness) problem: variable selection with microarray data can lead to many solutions that have similar prediction errors, but that share few common genes [1,6-9]. Choosing any one particular set of genes without being aware of the variability in solutions can lead to a false sense of certainty in the selected set.

From a users' perspective, an ideal tool should also be user friendly and provide additional resources to ease the interpretation of results [10]. Web-based tools are an excellent platform as they do not require software installation or upgrades from the user. In addition, web based tools, can be designed to allow easy access to information such as Gene Ontology terms, the UCSC and Ensembl databases, KEGG and Reactome pathways, or PubMed references, thus enhancing the biological interpretation of results [11]. Moreover, web-based tools, if implemented appropriately, can harvest computational resources rarely available to most individual users [10], including the increasing availability of multicore processors and easily accessible clusters made with off-the-shelf components [12]. Currently, the major opportunities for improved performance as well as the ability to analyze ever larger data sets do not lie in faster CPUs but in being able to use parallel and distributed computing to exploit multi-core servers and clusters [13,14]. In addition to providing a benefit to the end user (decreased execution time), tools that combine parallelization with web-based programming are important methodological developments.

Finally, a tool that fulfills the above requirements is of much greater relevance if it makes its source code available under an open-source license. Source code availability allows the research community to experiment with, and improve upon, the method and fix bugs, encourages reproducible research, allows to verify claims by method developers, makes the international research community the owner of the tools needed to carry out its work and, thus, creates the conditions for swift progress upon previous work, concerns of particular importance in bioinformatics [15,16].

We have developed GeneSrF and varSelRF (a web-based application and R package, respectively), that satisfy the above requirements. The only available web-based tools with similar scope are M@CBETH [17] and Prophet [18]. These tools, however, do not examine the multiplicity problem, cannot benefit from multicore processors or computing clusters, and do not make source code available. M@CBETH, in addition, is restricted to two-class problems and does not focus on the gene selection problem. Prophet, in turn, does not seem to solve satisfactorily the biased error rate problem (it reports the error rate as that of the classifier with smallest cross-validated error rate, without evaluating the error rate of the rule itself).

## Implementation

**Figure 1:**
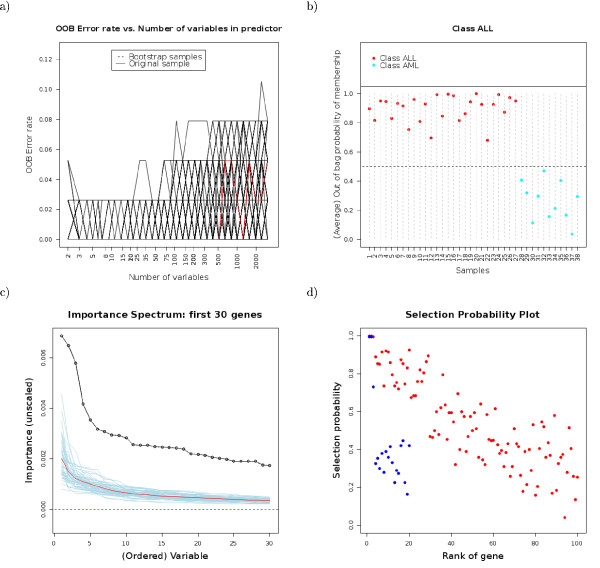
**Example output**. Some figures from the output of the web-based application (see [24]). a) Out-of-bag error rate vs. the number of genes in the class prediction model, for both the complete, original data set (red line) and the 200 bootstrap samples (black lines). These figures can help identify the best number of genes in the class prediction model. It seems, that we can do fairly well using just 2 genes in our model. This is the conclussion we reach both with the complete, original data set and the bootstrap samples. b) Probability of class membership of each sample, from out-of-bag samples (i.e., bootstrap runs where the sample was not included in the training group). Most samples are well classified, specially those from class ALL (their average out of bag probability of membership in their true class is larger than 0.75). c) Importance spectrum plots can help decide on the number of "relevant variables": we compare the variable importance plots from the original data with variable importance plots that are generated when the class labels and the predictors are independent (class labels are randomly permuted). In this case the first 30 variables have importances well above those from sets with randomly permuted class labels. d) Selection probability plots: for each of the top ranked genes from the original sample, the probability that it is included among the top ranked k genes (blue: k = 20; red: k = 100) from the (200) bootstrap samples. Thus, these plots can be a measure of our confidence in the stability of choosing a number of k ranked genes. In this case, with k = 20 only the two or three most important genes are repeatedly chosen among the best 20. If we select the first 100 genes, the 30 best ranked ones appear at least in 75% of the bootstrap samples.

The core statistical functionality is provided by the varSelRF package for R [19]. This package implements the procedure in [1] for gene selection using random forests, building upon the randomForest package [20], an R port by A. Liaw and M. Wiener of the original code by L. Breiman and A. Cutler. We use MPI [21] for parallelization via the R-packages Rmpi [22] by H. Yu, and Snow [23] by L. Tierney, A. J. Rossini, Na Li and H. Sevcikova. In the web-based application, the CGI, initial data validation, and the setting-up and closing of the parallel infrastructure (booting and halting the LAM/MPI universes) is implemented with Python. Our installation runs on a cluster of 30 nodes, each with two dual-core AMD Opteron processors (see Figure 1 for details).

The input for the web-based application are either plain text files, or files that come from other tools of the Asterias suite [12]. GeneSrF has been running in production use for over a year. Further documentation and examples for the web-based application are available from its on-line help, and for the R package from the standard R documentation system. A fully commented example of the output is provided in the on-line help [24]. Sample output is shown in Figure 1. Bug-tracking and additional tests are available from Bioinformatics.org and The Launchpad.

## Benchmarks and run time

**Figure 2:**
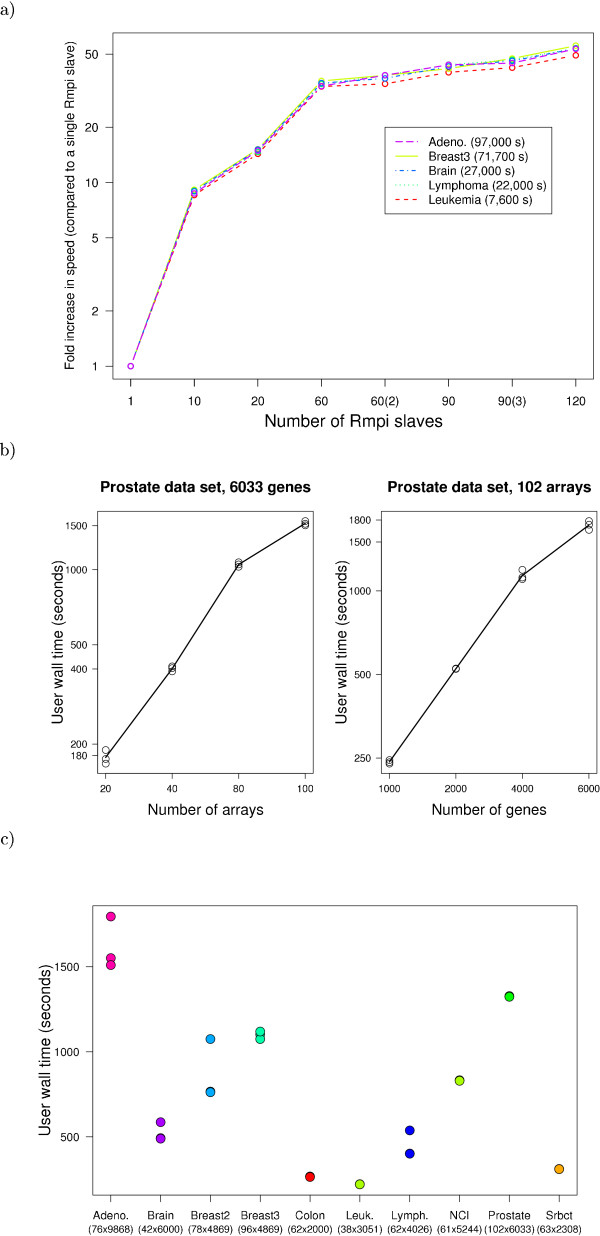
**Benchmarks and run time**. a) Fold increase in speed from parallelization. Ratios of the user wall time of execution of the R code (varSelRFBoot without previous model fit) between a run with a single Rmpi slave and runs with different numbers of Rmpi slaves (the number of simultaneously executing R processes) for five data sets (see [1] for details). In the legend, in parentheses the user wall time of the execution with a single Rmpi slave for each data set. In all cases (except "1", "60(2)", and "90(3)") there were four Rmpi slaves per node. The timings were obtained in an otherwise idle cluster with 30 nodes, each with two dual-core AMD Opteron 2.2 GHz CPUs and 6 GB RAM, running Debian GNU/Linux and a stock 2.6.8 kernel, with version 7.1.2 of LAM/MPI and version 2.1.4 (patched) of R. The values for "60(2)" refer two a configuration with 2 slaves per node (recall that a node with two dual core CPUs is not identical to a node with 4 CPUs), and the value "90(3)" to a configuration with 3 slaves per node. b) Scaling of user wall time. User wall time as a function of number of arrays and number of genes when executing the R function varSelRFBoot without previous model fit. Shown are three replicate runs. In each run, the arrays and genes are selected randomly from the complete original data set. Further details about the Prostate data set from [1]. Hardware and software as above. We used 4 Rmpi slaves per node (and, thus, a total of 120 slaves). c) User wall time of the web-based application. User wall time for complete runs (i.e., including upload of files and return of complete HTML page) for ten different data sets (see details in [1]). Under the name of each data set, the number of arrays and the number of genes are indicated. For each data set, three replicate runs were conducted. Hardware and software configuration as above, with the default settings for the web-based application (4 Rmpi slaves per node, and thus a total of 120 slaves).

The parallelization has been implemented over bootstrap resamples. The speedups achieved by parallelizing are shown in Figure 2a), where we plot the fold increase in speed achieved by increasing the number of Rslaves (concurrently executing R processes). Parallelization makes a dramatic difference in speed for all the data sets shown. Up to 20 Rmpi slaves, the increases in speed are almost linear with number of slaves. Beyond 20 slaves, speed increases are slower with number of slaves: as is known from the parallelization literature [21,25], in addition to number of CPUs other factors can become limiting, in our case most likely bandwith and latency of inter-node communication, and potential bottlenecks from memory and cache in nodes made of dual-core processors [26].

The scaling of user wall time of the R code (varSelRFBoot) with number of arrays and number of genes is shown in Figure 2b), with the default parallelization scheme and with a data set that allows for exploring a range of numbers of arrays and genes. User wall time increases approximately linearly with the number of arrays and number of genes over a realistic range of arrays and genes (e.g., when we double the number of arrays from 40 to 80 the user wall time increases by a factor of slightly over 2).

The run time for the web-based application for a wide range of data sets is shown Figure 2c). These timings include the time needed to upload the files (and thus can be affected by internet connection speed) and to prepare and return to the user the final figures. Note that in most cases the complete analysis is finished within 20 minutes.

Scripts for timing experiments are included with the source code (directory "Benchmarks").

## Results and discussion

Our procedure is explicitly targeted to select very small sets of genes, and has been shown [1] to have a classification error rate on-par with other, state-of-the-art, classification procedures. Additionally, our programs allow the exploratory usage of random forest for identifying large subsets of genes potentially relevant for class prediction. In contrast to other tools, such as M@CBETH [17], we are not restricted to two-class problems.

To avoid underestimating the error rate of the classification procedure, we use the bootstrap (the 0.632+ approach of [27]). As in [1], we bootstrap the complete procedure, including selecting the classifier with minimal out-of-bag error rate (thus, this is a "full" or "double" bootstrap procedure, sensu [5]), and thus our estimates of error rate are not affected by selection biases. This contrasts, for instance, with Prophet [18], where the error rate reported is that of the classifier with the smallest cross-validated error rate. Based upon the bootstrap results, we also show the average out-of-bag predictions for each sample, allowing to easily asses poorly predicted samples and potential outliers. There are other tools available for performing cross-validation and bootstrap of classification methods, such as the R package ipred [28] by A. Peters and T. Hothorn, the BioConductor package MCRestimate [29] by M. Ruschhaupt, U. Mansmann, P. Warnat, W. Huber and A. Benner, specifically targeted to computing misclassification error rates combining the gene selection and classification steps, or the caGEDA web application [10] that incorporates bootstrap, leave-one-out, and random resampling validation of several classifiers. Our approach, however, has been tailored to our own variable selection procedure and has been parallelized. A unique feature of GeneSrF and varSelRF are their emphasis on examining possible multiple solutions.

Since we obtain 200 resamples in the process of bootstrapping (see above) there is little added computational cost to providing analysis of stability and multiplicity of solutions. We report the number of genes selected and the identity of the individual genes selected in the original sample and the 200 bootstrap runs, including frequencies of every gene selected in the solutions. Moreover, the biological interpretation of the results is enhanced by the access to additional information. If the input file contains gene identifiers for either human, mouse, or rat genomes (in the form of Affymetrix IDs, Clone IDs, GenBank Accession numbers, Ensembl Gene IDs, Unigene clusters, or Entrez Gene IDs), for each gene in the results, the web-based application provides a link to IDClight [11], which allows the user to obtain additional information, including mapping between gene and protein identifiers, PubMed references, Gene Ontology terms, and KEGG and Reactome pathways. The multiple solutions can be further studied by sending sets of selected genes to our tool PaLS [30] to examine PubMed references, Gene Ontology terms, KEGG pathways, or Reactome pathways that are common to a user-selected percentage of genes or lists (bootstrap solutions). A fully commented example of the output is provided in the on-line help [24].

Finally, GeneSrF is one of the very few tools for the analysis of gene expression data that uses parallelization and, as far as we know, the only web-based tool to use parallelization for gene selection and classification. This is an important methodological novelty, as we can no longer expect that increases in single-CPU speed will allow us to analyze larger data sets in shorter time: the rate of increase in CPU speed has slowed down considerably in the last five years but, in contrast, increasing numbers of CPU cores (either in individual machines – including laptops – or via off-the-shelf computing clusters) are becoming much more affordable [13,14]. Thus, further decreases in user wall time (time to wait for a result) and ability to tackle more complex problems will depend on our ability to use parallel, distributed, and concurrent programming. GeneSrF therefore represents a case example on combining parallel computing with a user-friendly web-based application for the analysis of gene expression data and, by making the full source code available, allows other researchers to build upon our developments.

Future work focuses on extending the software to use random forest-related techniques applicable to heterogeneous types of variables such as addition of categorical data [31] and other clinical information. As well, we are exploring alternative mechanisms and languages for parallelizing and distributing computations, and we are rewriting most of the code using Pylons [32], a Python web framework, to try to simplify installation of the web-based application. Installation now involves several steps (see [33]), and the most time consuming are setting up and verifying the LAM/MPI environment, and using the correct paths in files involved in controlling the MPI environment and executing and controlling R.

## Conclusion

varSelRF and GeneSrF implement a validated method for gene selection and provide bootstrap estimates of classification error rate, take advantage of computing clusters and multicore processors, and encourage careful examination of the multiplicity of solutions problems. Thus, these are both useful tools for applied biomedical researchers using microarray and gene expression data, and represent unique methodological developments in the area of web-based gene expression analysis tools.

## Availability and requirements

For GeneSrF:

**Project name: **GeneSrF

**Project home page: **

**Operating system: **Platform independent (web-based application)

**Programming language: **R, Python

**Other requirements: **A web browser.

**License: **None for usage. Web-based code: Affero GPL (open source).

**Any restrictions to use by non-academics: **None.

For varSelRF:

**Project name: **varSelRF

**Project home page: **

**Operating system: **Linux, Unix

**Programming language: **R, Python

**Other requirements: **LAM/MPI

**License: **GNU GPL

**Any restrictions to use by non-academics: **None

## Abbreviations

CGI, Common Gateway Interface; GO, Gene Ontology; KEGG, Kyoto Encyclopedia of Genes and Genomes; LAM, Local Area Multicomputer; MPI, Message Passing Interface.
